# Mortality Effects of a Copper Smelter Strike and Reduced Ambient Sulfate Particulate Matter Air Pollution

**DOI:** 10.1289/ehp.9762

**Published:** 2007-01-04

**Authors:** C. Arden Pope, Douglas L. Rodermund, Matthew M. Gee

**Affiliations:** Department of Economics, Brigham Young University, Provo, Utah, USA

**Keywords:** air pollution, copper smelter, mortality, natural experiment, particulate matter, sulfates

## Abstract

**Background:**

Numerous studies have reported associations between fine particulate and sulfur oxide air pollution and human mortality. Yet there continues to be concern that public policy efforts to improve air quality may not produce actual improvement in human health.

**Objectives:**

This study retrospectively explored a natural experiment associated with a copper smelter strike from 15 July 1967 through the beginning of April 1968.

**Methods:**

In the 1960s, copper smelters accounted for approximately 90% of all sulfate emissions in the four Southwest states of New Mexico, Arizona, Utah, and Nevada. Over the 8.5-month strike period, a regional improvement in visibility accompanied an approximately 60% decrease in concentrations of suspended sulfate particles. We collected monthly mortality counts for 1960–1975 and analyzed them using Poisson regression models.

**Results:**

The strike-related estimated percent decrease in mortality was 2.5% (95% confidence interval, 1.1–4.0%), based on a Poisson regression model that controlled for time trends, mortality counts in bordering states, and nationwide mortality counts for influenza/pneumonia, cardiovascular, and other respiratory deaths.

**Conclusions:**

These results contribute to the growing body of evidence that ambient sulfate particulate matter and related air pollutants are adversely associated with human health and that the reduction in this pollution can result in reduced mortality.

Numerous epidemiologic studies have reported associations between ambient particulate matter (PM) air pollution and mortality (Pope and Dockery 2006). Most of these studies have evaluated associations between daily mortality counts and short-term (day-to-day) changes in pollution concentrations using either time-series (Aga et al. 2003; Analitis et al. 2006; Dominici et al. 2003; Samet et al. 2000; Schwartz 2000) or case–crossover (Schwartz 2004) study designs. Various population-based cross-sectional (Evans et al. 1984; Lave and Seskin 1970; Özkaynak and Thurston 1987) and prospective cohort (Dockery et al. 1993; Jerrett et al. 2005; Laden et al. 2006; Pope et al. 2002; Pope et al. 1995) studies have also evaluated mortality associations with long-term (years to decades) differences in average concentrations across different metropolitan areas or communities. These studies found mortality risk to be consistently associated with concentrations of PM_2.5_ (a common indicator of fine particulate matter including particles with an aerodynamic diameter ≤ 2.5 μm cut point, which include most sulfate particles). Only a few studies of natural experiments have evaluated large-scale mortality effects associated with sharp, prolonged reductions in air pollution. Reductions in mortality accompanied substantial reductions in PM concentrations associated with the closure of a steel mill in Utah Valley, Utah (Pope et al. 1992), the banning of coal burning in Dublin, Ireland (Clancy et al. 2002), and a reduction in sulfur oxides through the imposition of fuel restrictions in Hong Kong (Hedley et al. 2002).

In the present study we explored an interesting natural experiment associated with a nationwide copper smelter strike from 15 July 1967 through early April 1968 (Cole 1969; Rowland and Greenspoon 1968). The strike had an especially large impact on four Southwest states with copper smelters: New Mexico, Arizona, Utah, and Nevada. In the 1960s, copper smelters accounted for approximately 90% of all sulfate emissions in these Southwest states. As reported by Trijonis and Yuan (1978) and Trijonis (1979), the 1967–1968 strike led to measurable regional reductions in suspended sulfate PM and increases in visibility throughout the Southwest states. Over the 8.5-month strike period, a regional improvement in visibility accompanied an approximately 60% (~ 2.5 μg/m^3^) decrease in concentrations of suspended sulfate particles. The primary objective of this study is to retrospectively analyze the mortality data and evaluate the evidence of strike-related reductions in mortality.

## Methods

### Study area

**Figure 1:**
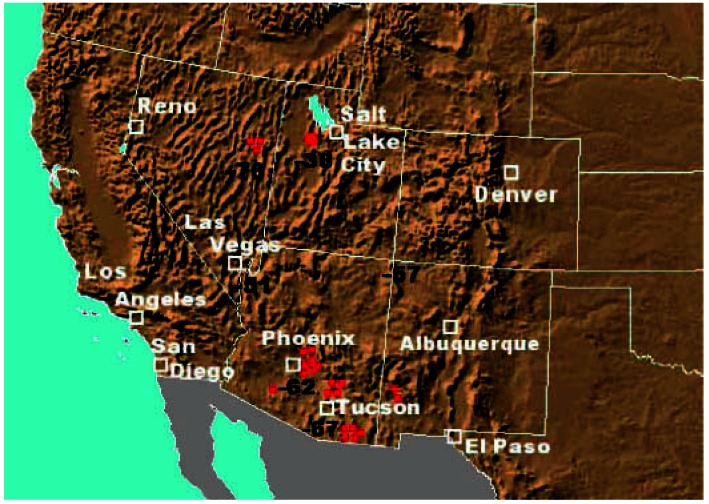
Locations of copper smelters (red circles) relative to major population centers (white boxes). Numbers indicate seasonally adjusted percent changes in sulfate concentrations during the smelter strike.

The primary study area in this analysis included the four Southwest states most influenced by the copper smelter strike: New Mexico, Arizona, Utah, and Nevada (Figure 1). As reported and analyzed by Trijonis (1979), visibility data in these four states were collected from urban airports, suburban/nonurban airports, urban NASN (National Air Surveillance Network) sites, and nonurban NASN sites. Phoenix and Tucson, Arizona; Las Vegas, Nevada; Salt Lake City, Utah; and Albuquerque, New Mexico, were the population centers in the states most affected by the strike. Colorado was not included because of the lack of copper smelters, the distance of its population centers from the smelter locations, and the insignificant change in air quality in the Denver area during the strike period (Trijonis 1979). Despite El Paso’s close proximity to smelters, Texas was not included because El Paso represented a very small portion of the state’s population.

### Mortality data

**Figure 2:**
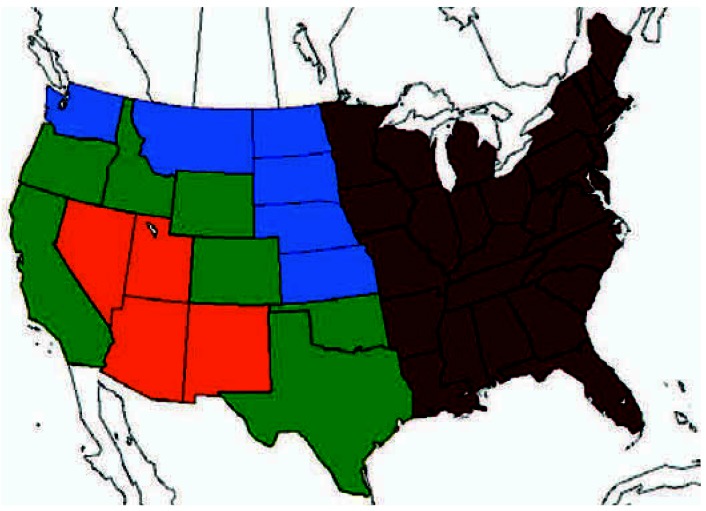
United States divided into four regions: four Southwest states (orange), bordering states (green), neighboring states (blue), and eastern states (red).

We collected mortality data from the National Center for Health Statistics’ yearly mortality reports of the United States from 1960 to 1975 (National Center for Health Statistics 1964–1979). Monthly mortality counts for the four Southwest states, both individually and combined, were compiled. The United States was also divided into four additional regions (rest of United States, Eastern states, neighboring states, and bordering states) according to their proximity to the four Southwest states (Figure 2). Monthly mortality counts for each of these four regions were compiled. Nationwide monthly mortality counts for several specific causes of death were also compiled. Because *International Classification of Diseases* (ICD) codes changed in January 1968 from the 7th [World Health Organization (WHO) 1957] to the 8th Revision (WHO 1967), ICD-7 codes were used for 1960–1967 and ICD-8 codes were used for 1968–1975. Six nationwide cause-of-death mortality variables were created: *a*) influenza/pneumonia 1960–1967 (ICD-7 codes 480–483, 490–493); *b*) influenza/pneumonia 1968–1975 (ICD-8 codes 470–474, 480–486); *c*) cardiovascular disease 1960–1967 (ICD-7 codes 400–468, 331, 332); *d*) cardiovascular disease 1968–1975 (ICD-8 codes 390–458); *e*) respiratory system 1960–1967 (ICD-7 codes 470–527); and *f* ) respiratory system 1968–1975 (ICD-8 codes 460–519).

### Analysis

We plotted mortality counts for the four Southwest states over time and over mortality counts for Eastern states, neighboring states, and bordering states. We also analyzed variation in death counts using 12 different Poisson regression models for the four Southwest states as a region as well as for each Southwest state individually. All of the models controlled for time trends in mortality. Because of potential nonlinearities in the time trends, the models included spline smoothers of time with 1–3 degrees of freedom using generalized cross-validation to select the best fit. To further control for non-event-related regional variability in mortality, models 1–4 included total mortality counts from all other U.S. states, the Eastern U.S. states, neighboring states, and bordering states (Figure 2), respectively. Models 5–8 were the same as models 1–4 except that the two variables for nationwide monthly mortality counts for influenza/pneumonia (using ICD-7 and ICD-8 codes for relevant time period) were also added to the models to control for the effects of a nationwide epidemic or other regional epidemics. Models 9–12 most aggressively controlled for U.S. non-event-related mortality trends by including all six nationwide cause-of-death variables (influenza/pneumonia, cardiovascular disease, and other respiratory deaths, as discussed above).

Because the strike began in the middle of July and because there may be a lagged pollution effect on mortality, we created three strike period indicator variables to account for different possible lagged effects: *a*) July 1967–March 1968 (all strike months including the first month that was only partially effected by the strike), *b*) August 1967–March 1968 (only the full strike months), and *c*) August 1967–April 1968 (full strike months plus 1-month lag). Statistical analyses were conducted using R and SAS software estimating the cubic spline smooths using the “gam” function in the MGCV package (Wood 2003; Wood 2004) in R (R Development Core Team 2006) and the “spline” function using PROC GAM in SAS (release 9.1.3; SAS Institute, Inc., Cary, NC).

## Results

**Figure 3:**
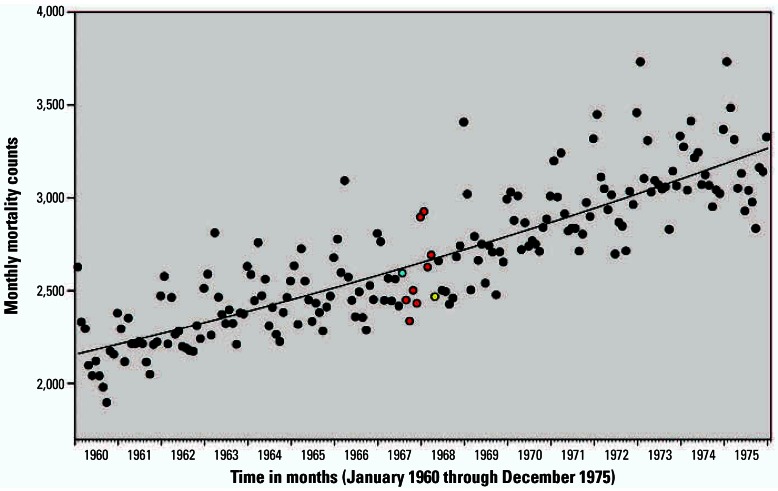
Monthly mortality counts for the four Southwest states plotted over time. The blue dot indicates the month when strike began mid-month (July 1967). Red dots indicate full strike months (August 1967–March 1968). Yellow dot indicates first end-of-strike month (April 1968).

**Figure 4:**
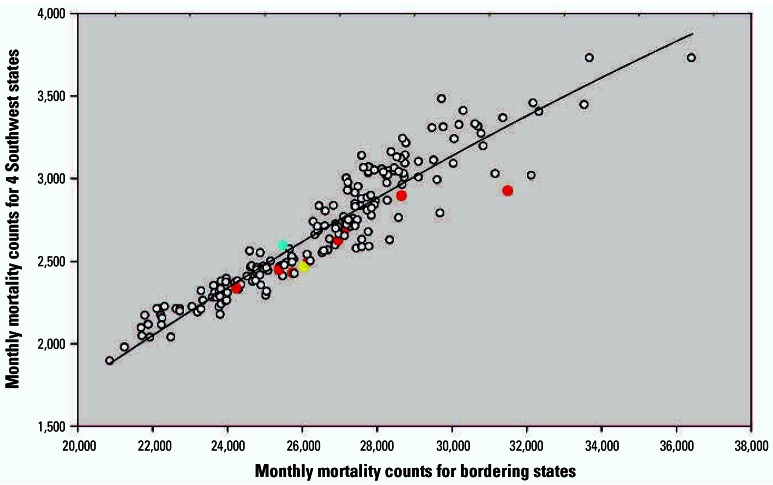
Monthly mortality counts for the four Southwest states plotted over monthly mortality counts for bordering states. The blue dot indicates the month when strike began mid-month (July 1967). Red dots indicate full strike months (August 1967–March 1968). Yellow dot indicates first end-of-strike month (April 1968). The line is a quadratic regression line fit through the data.

Monthly mortality counts from the four Southwest states plotted over time are presented in Figure 3. Characteristic nonstationary Poissonian variability, time trend, and seasonal components of mortality counts are observable, but the effect of the strike period is small compared with other sources of variability. Figure 4 plots monthly mortality counts in the four Southwest states over monthly mortality counts for bordering states, which presumably have similar time trends, seasonality, and other regional similarities. Although the mortality counts in the four Southwest states were consistently below what would be expected based on the mortality trends of bordering states, the relative reductions in mortality were small.

**Figure 5:**
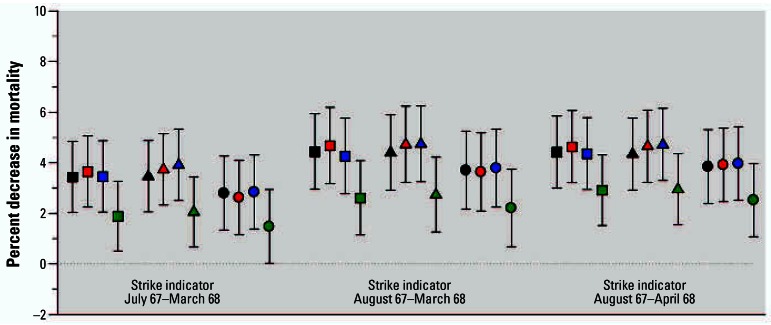
Estimated smelter strike–related decreases in mortality (and 95% CIs) for all four Southwest states using alternative models. Black, red, blue, and green symbols indicate the inclusion of total mortality counts from all other U.S. states, eastern U.S. states, neighboring states, or bordering states, respectively. Squares, triangles, and circles indicate models that additionally included none, just influenza/ pneumonia, or all six nationwide cause-of-death variables, respectively.

**Figure 6:**
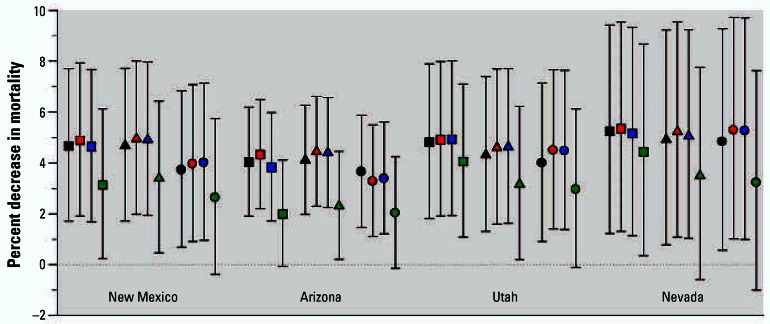
State-specific estimated decreases in mortality (and 95% CIs) associated with August 1967–April 1968 strike indicator period using alternative models. Black, red, blue, and green symbols indicate the inclusion of total mortality counts from all other U.S. states, eastern U.S. states, neighboring states, or bordering states, respectively. Squares, triangles, and circles indicate models that additionally included none, just influenza/pneumonia, or all six nationwide cause-of-death variables, respectively.

Small but statistically significant strike-related decreases in mortality were observed based on the Poisson regression models. Figure 5 presents the estimates of the percent decrease in mortality [and 95% confidence intervals (CIs)] in the four Southwest states associated with the three different strike indicator periods for the 12 different Poisson regression models. Strike period mortality effect estimates indicated a decrease in mortality of approximately 1.5–4.0% depending on the model and strike period indicator used. For example the estimated percent decrease in mortality was 2.5% (95% CI, 1.1–4.0%), based on a model that used a strike period indicator for full strike months plus a 1-month lag (August 1967–April 1968) and that controlled for time trends, mortality counts in bordering states, and nationwide monthly mortality counts for influenza/pneumonia, cardiovascular, and other respiratory deaths. As can be seen in Figure 6, similar patterns in the effect estimates were observed for each of the four Southwest states individually.

**Figure 7:**
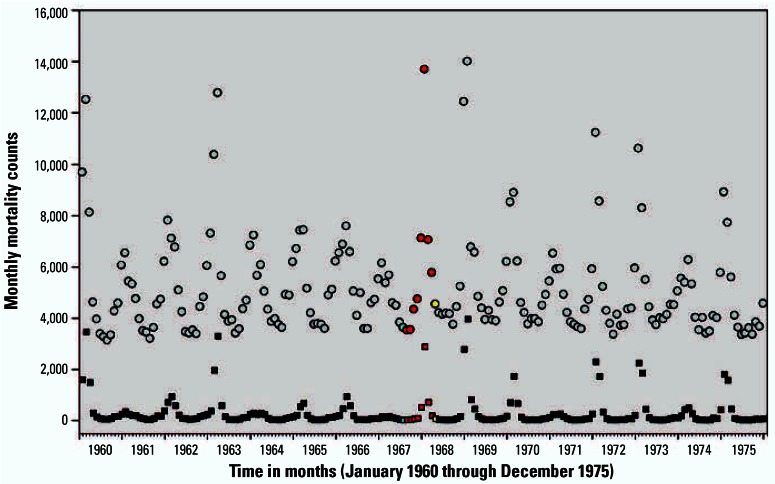
Monthly mortality counts for influenza (squares) and influenza/pneumonia (circles) in the United States plotted over time. Blue indicates the month when strike began mid-month (July 1967). Red indicates full strike months (August 1967–March 1968). Yellow indicates first end-of-strike month (April 1968).

Figure 7 presents plots of nationwide monthly mortality counts for influenza and pneumonia. The Hong Kong pandemic of 1968–1969 (Kilbourne 2006) along with other periods of elevated influenza/pneumonia, are observable. However, as demonstrated by the results presented in Figures 5 and 6, the strike period effect estimates were not highly sensitive to controlling for nation-wide monthly mortality counts for influenza/pneumonia, cardiovascular, and other respiratory deaths. The effect estimates were also not sensitive to whether the models controlled for mortality trends in the rest of the United States, eastern U.S. states, or neighboring states, but were consistently smaller when counts for the bordering states were included. Somewhat larger effect estimates were observed when the strike indicator did not include the first partial month of the strike and included a 1-month lag.

## Discussion

The 8.5-month copper smelter strike in 1967–1968 provided a unique and interesting natural experiment to explore the mortality effects of air pollution. Relative to the natural experiment studies of Utah Valley (Pope et al. 1992) and Dublin (Clancy et al. 2002), the copper smelter strike resulted in smaller reductions in fine particulate pollution, but the reductions occurred over a much larger geographic region. This retrospective analysis of available mortality data suggests that the approximately 60% reduction in regional sulfate concentrations (~ 2.5 μg/m^3^) associated with the copper smelter strike produced a small but measurable reduction in mortality (1.5–4.0%). The effect estimates were reasonably stable and robust to controlling for time trends, mortality trends in other areas of the United States, and nationwide monthly mortality counts for influenza/pneumonia, cardiovascular, and other respiratory deaths. The effect estimates were consistently reduced when mortality counts in bordering states were included in the models. Mortality counts from bordering states are more likely to have similar mortality trends and seasonality and other regional similarities, as well as common influences of regional communicable disease. Therefore, the use of mortality counts from bordering states as a control variable in the regression may be preferable to including mortality counts from more distant areas of the United States. However, because the air quality of the bordering states may also have been somewhat improved because of the smelter strike, using counts in the bordering states may result in overcontrolling and underestimating the mortality effect.

The estimated strike-related reduction in mortality of approximately 1.5–4.0% is consistent with what would be expected given the reduction in ambient concentrations of sulfate particles and estimated mortality effects from the relevant literature. Based on data presented by Trijonis and Yuan (1978), the average, seasonally adjusted, regional strike-related reduction in concentrations of sulfate particles was approximately 2.5 μg/m^3^. Based on reported mortality reductions from the natural experiment studies of Utah Valley (Pope et al. 1992) and Dublin (Clancy et al. 2002), a 2.5 μg/m^3^ reduction in PM_2.5_ concentrations would result in approximately a 0.8–1.1% decline in overall mortality, depending on assumptions about PM conversion factors and methods of extrapolation (Pope and Dockery 2006). The estimated percent change in mortality associated with a 2.5-μg/m^3^ change in PM_2.5_ from long-term prospective cohort studies would equal approximately 1.5% based on nationwide American Cancer Society studies (Pope et al. 1995, 2002) and up to 4.0% based on the Harvard Six Cities (Dockery et al. 1993; Laden et al. 2006) and American Cancer Society, Los Angeles intrametropolitan area studies (Jerrett et al. 2005). In a recent U.S. Environmental Protection Agency (EPA) expert elicitation (U.S. EPA 2006) on the assessment of the concentration response relationship between PM_2.5_ exposure and mortality, multiple experts suggest that a long-term 1-μg/m^3^ decrease in PM_2.5_ would lead to a mean reduction in mortality of 0.4–2.0% with credible estimates ranging from 0 to 3.0%—comparable with effect estimates observed in this analysis.

There are several different reasons to expect different mortality effects from this natural experiment versus other relevant particulate air pollution studies. Given the ubiquitous regional exposure and the high propensity of sulfate particles to penetrate indoors (Sarnat et al. 2000, 2006; Suh et al. 1993), the smelter strike–related reduction in ambient air pollution may reflect relatively large reductions in personal exposures. Specific copper smelter–related sulfate particles and related co-pollutants may have different relative toxicities. Although detailed data on changes in levels of transition metals are not available, it is likely that there were important reductions in transition metals associated with the temporary closure of the copper smelters. There is evidence that fine particles with proportionally high levels of transition metals are relatively more toxic (Ghio 2004; Ghio and Cohen 2005). The strike may have also caused an exodus of smelter workers to neighboring regions, which may have artificially lowered the death counts in the four affected Southwest states during the strike period. Evidence that this is not the explanation for the strike-related reduction in mortality is provided by models that adjusted for mortality counts in bordering states. Adjustment for bordering state mortality counts reduced the strike effect estimates, whereas the opposite would be expected if the workers left the affected states to increase disproportionately the population of the bordering states.

A limitation of this study is that other events or factors that occurred approximately concurrent with the smelter strike may have caused the reduction in mortality in these four Southwest states and were not adequately controlled for by time trend variables, mortality counts in neighboring areas, and nationwide mortality counts for influenza/pneumonia, cardiovascular, and other respiratory deaths.

Another limitation of this analysis is the lack of spatial resolution with regard to the pollution and mortality data. For example, the results of this analysis are generally consistent with the interpretation that the copper smelter strike resulted in an 8.5-month reduction in fine sulfate PM, contributing to a concurrent reduction in mortality. However, one finding that is somewhat inconsistent with this interpretation is that similar strike-related mortality associations were observed in all four Southwest states, including New Mexico. However, based on analysis presented by Trijonis (1979), changes in visibility and sulfate concentrations in New Mexico were not as largely affected by the strike as the other three states. Another limitation of this analysis comes from the lack of available monthly data on cause of mortality for each state. These data would allow for an analysis to be conducted specifically on the effect of PM on cardiorespiratory related deaths.

Evidenced by the elevated effect estimate when the lagged month of April was included as part of the strike period indicator, this analysis suggests a lag effect of a few weeks to a month. There is no evidence that the lag effect lasted longer than 1 month.

In conclusion, the results of this study add to the growing body of evidence that ambient sulfate particulate matter and related air pollutants are associated with adverse human health effects and that the reduction in this pollution can reduce mortality. These results suggest that the approximately 60% reduction in regional sulfate concentrations (~ 2.5 μg/m^3^) associated with the copper smelter strike produced improvements not just in visibility but also in small but measurable reductions in mortality.

## Correction

The concentration of sulfate particles noted in the manuscript originally published online as 1–1.5 μg/m^3^ has been corrected to ~ 2.5 μg/m^3^ in the introduction and the “Discussion.” Also in the “Discussion,” the decline in overall mortality originally was 0.2–0.7% and the estimated percent changes in mortality were 0.6–0.9% and 1.7–2.6%. They have been corrected here to 0.8–1.1%, 1.5%, and 4.0%, respectively.
